# Selection on Sex Cells Favors a Recombination Gender Gap

**DOI:** 10.1371/journal.pbio.0030099

**Published:** 2005-02-22

Males and females of the same species can be strikingly different. Peacocks strut around with flashy feathers to attract mates, while peahens blend into their surroundings with more subdued colors. But differences are not always as obvious or easily explainable as in this classic example. Even the amount of genetic reshuffling that goes on during egg and sperm production differs between males and females in most species. An evolutionary reason for this has eluded researchers since the phenomenon was originally discovered in fruitflies, Chinese silk worms, and amphipods almost 100 years ago.

Genetic diversity among organisms is promoted when genetic information is rearranged during meiosis, the cell division process that yields sperm and eggs (generically called gametes). During this genetic reshuffling, chromosome pairs overlap, forming structures called chiasmata (“crosses” in Greek), and physically recombine. This process does not just create diversity, it is also an example of diversity—recombination rates vary across chromosomes, sexes, and species.[Fig pbio-0030099-g001]

**Figure pbio-0030099-g001:**
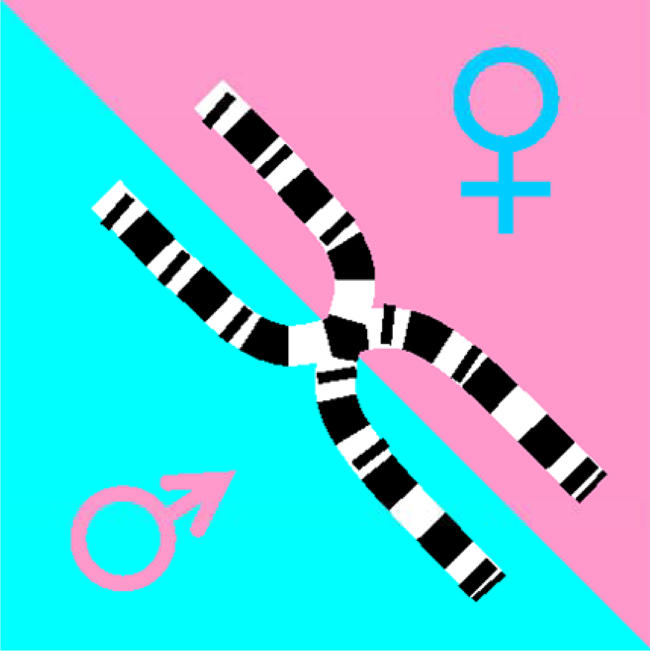
Male and female recombination rates differ

An early 20th century hypothesis to explain the sex difference in recombination proposed that recombination is restrained within a pair of unlike sex chromosomes (X and Y, for example) and that the suppression spills over to the rest of the chromosomes. Under this idea, the sex with dissimilar sex chromosomes (XY instead of XX, for example) should be the one with the least amount of recombination in all chromosomes. But that is not always the case. Some hermaphroditic species of flatworms, for example, lack sex chromosomes altogether but still display marked differences in male and female recombination rates. In one salamander genus, more reshuffling unexpectedly occurs in the sex with two different sex chromosomes.

In a new study analyzing an updated dataset of 107 plants and animals, Thomas Lenormand and Julien Dutheil bolster the argument against the recombination suppression hypothesis by showing that in species with sex chromosomes, the sex with two dissimilar sex chromosomes doesn't necessarily have a reduced recombination rate. Additionally, they found that, as a trait, the sex difference in recombination rate is not a lot more similar between two species in the same genus than between two species in different genera, suggesting that the difference evolves quickly.

An alternative hypothesis suggests that sexual selection might play a role in recombination differences. Reproductive success among males is often highly influenced by selection, so mixing up successful genetic combinations in males could be evolutionarily counterproductive. But in past studies, sexual selection was not related to variation in recombination rates.

Putting a new twist on this hypothesis, Lenormand and Dutheil realized that selection was not necessarily limited to the adult stage and that differences in selection among eggs or sperm might help account for recombination differences between the sexes. The authors reasoned that more opportunity for selection on sperm than egg should correspond to less recombination during sperm than egg production (and vice versa), consistent with the idea that genetic combinations surviving selection should remain more intact in the sex experiencing the strongest selection at the gametic stage.

Though male gametes might be expected to be under stronger selection in many species, in true pines it seems to be the female gametes. The ovules compete with each other for resources over an entire year before being fertilized, and, indeed, from the dataset analysis, ovule production involves low recombination rates compared with male pollen in this group. In males, the opportunity for pollen competition was indirectly estimated using self-fertilization rates. The authors assumed that pollen grains competing for ovules of a self-fertilizing plant would be genetically similar and therefore experience less selection. Again, in the analysis, low selection correlated with less recombination in female gamete production, as predicted.

Is selection among eggs and sperm the evolutionary force generating sex-based variation in genetic shuffling? By demonstrating that differences may be influenced by gamete selection in plants, this work has added clarity to otherwise contradictory observations.

